# Long-Term Outcomes Associated with the Use of a Soft, Partially Absorbable Transobturator Mid-Urethral Tape for the Treatment of Stress Urinary Incontinence

**DOI:** 10.3390/jcm14103572

**Published:** 2025-05-20

**Authors:** Ronen S. Gold, Jonatan Neuman, Menahem Neuman, Asnat Groutz

**Affiliations:** 1 Urogynecology Unit, Department of Obstetrics and Gynecology, Lis Women Hospital, Tel Aviv Medical Center, Tel Aviv University Medical School, Tel Aviv 6423906, Israel; agroutz@yahoo.com; 2 Medical School, Semmelweis University, 1085 Budapest, Hungary; neuman@gmail.com; 3 The Urogynecology Service, Assuta Medical Centers, Ben Gurion University Medical School, Tel Aviv 6971028, Israel; menahem.neuman@gmail.com

**Keywords:** complications, mid urethral sling, outcomes assessment, stress urinary incontinence

## Abstract

**Objectives:** To assess the long-term safety and efficacy of the Serasis^®^ inside-out transobturator midurethral sling (MUS), a partially absorbable soft tape for stress urinary incontinence (SUI). **Methods:** A cohort study of 146 consecutive women who underwent the Serasis^®^ MUS procedure from January 2013 to January 2014 was investigated. All patients had SUI as the main complaint. Patients with predominant urgency urinary incontinence (UUI) and stage III-IV pelvic organ prolapse were excluded. Clinical, intraoperative, and postoperative data were retrospectively retrieved from a computerized database. At 10 years postoperatively, a follow-up telephone survey was conducted. The patients were interviewed regarding tape-related complications, repeated SUI surgery, and decision regret or satisfaction. **Results:** All patients underwent the Serasis^®^ MUS procedure, most of whom also had concomitant colporrhaphies. The mean duration of surgery was 26.03 min, and the mean blood loss was 32.4 cc. All patients were discharged within a few hours after surgery or on the following day. No significant intraoperative or early postoperative complications were reported. Overall, 107 (73.3%) patients were available for the 10-year follow-up, 17 (15.9%) of whom reported symptoms of SUI, but only half of them underwent a repeated MUS. The rate of tape erosion was 1.9%, and no symptoms of tape-related pain were reported. Additionally, 10.3% of the patients were categorized as a subjective failure, most of whom considered persistent UUI as the main reason for dissatisfaction. **Conclusions:** The long-term outcomes of the transobturator Serasis^®^ MUS, a partially absorbable soft tape, are favorable and are associated with significantly fewer tape-related complications.

## 1. Introduction

Stress urinary incontinence (SUI) is a common condition in women, characterized by the involuntary leakage of urine during increased intra-abdominal pressure. Up to 40–50% of the adult female population is affected by SUI, with 18% of these individuals experiencing mixed urinary incontinence (MUI), which is a combination of SUI and urgency urinary incontinence (UUI) [1]. The main risk factors for SUI and MUI are vaginal deliveries, aging, and obesity [1,2]. It is expected that the prevalence of urinary incontinence will increase as the population ages and obesity rates rise. These high prevalence rates are associated with impaired quality of life and a significant social and economic burden [2,3].

The first-line management of SUI and MUI includes lifestyle modifications, such as weight reduction, smoking cessation, and pelvic floor muscle training. If initial conservative management fails, surgical intervention may be warranted [4]. Mid-urethral sling (MUS) is currently considered the standard surgical treatment for SUI [4,5,6,7]. A modified intravaginal slingplasty for the surgical treatment of SUI was first introduced by Ulmsten et al. in 1995 [8]. This minimally invasive procedure was proven to be both effective and safe and, over the past 30 years, has gained popularity and has undergone several modifications [9,10,11]. It can be performed using either a retropubic or transobturator approach, with both inside-out or outside-in techniques. Short- and medium-term follow-up studies have demonstrated no significant differences between the objective and subjective outcomes of the retropubic and transobturator approaches [4,5,6,7]. Less is known regarding the long-term safety and efficacy of the various modifications of MUS surgery [12,13,14,15,16,17,18,19]. Moreover, there have been concerns regarding the technique’s safety. In recent years, mesh-related complications such as erosion, pelvic pain, and dyspareunia have led to the ban of MUS in several countries [19,20].

**Figure 1:**
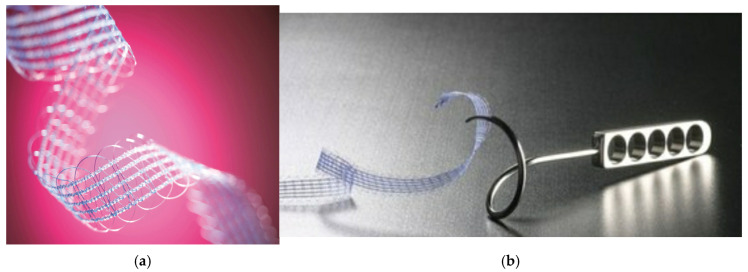
(**a**) SERASIS^®^ PA MR tape (Serag-Wiessner, Naila, Germany). (**b**) SERASIS^®^ TO Nahtinstrument reusable stainless steel guide and SERASIS^®^ PA Textile Implantate.

Serasis^®^ (Serag-Wiessner, Naila, Germany) is a MUS system with a reusable stainless steel guide and a soft, partially absorbable mesh implant. The mesh implant has large pores and is a bi-component material made from non-absorbable polypropylene and absorbable polyglycolic acid-caprolactone (PGACL). The PGACL is absorbed within 90–120 days, creating a soft fabric without sharp edges and potentially reducing tissue trauma and tape-related complications (Figure 1a,b). The tape may be inserted either via a transobturator or through a retropubic approach. Currently, no data are available regarding this potentially improved tape’s long-term safety and efficacy.

The objective of the present study was to assess the safety and long-term efficacy of the transobturator inside-out partially absorbable Serasis^®^ MUS in the treatment of SUI.

## 2. Materials and Methods

A cohort study of 146 consecutive women with SUI who underwent inside-out transobturator Serasis^®^ MUS from January 2013 to January 2014 was investigated. The Institutional Review Board approved the study protocol (ASMC 0020-24). All patients had simple, non-complicated SUI with no voiding dysfunction, dominant urgency incontinence, or significant pelvic organ prolapse.

Demographic, clinical, intraoperative, and postoperative data were retrospectively retrieved from a computerized database. Preoperative data included age, parity, severe comorbidities, prior pelvic floor surgeries, and lower urinary tract symptoms (LUTS). Preoperative evaluation included a physical and pelvic examination to assess pelvic organ prolapse (POP) and urethral mobility, a stress test to confirm SUI, and an ultrasound to exclude significant post-void residual urine. The severity of POP was categorized into stages 0 through IV based on the POP-Q classification system. Patients with predominant UUI and stage III-IV POP were excluded. All patients underwent MUS using the partially absorbable Serasis^®^ tape. A concomitant native-tissue repair was performed for patients with either anterior (cystocele) and/or posterior (rectocele) pelvic organ prolapse (POP). A single experienced urogynecologist performed all surgical procedures.

Intraoperative data included the duration of surgery (minutes), estimated blood loss (cc), and surgical complications. Early follow-up assessment was carried out one month and four months postoperatively. The postoperative evaluation included anatomical and functional cure rates, pain, dyspareunia, and LUTS. In September 2023, one of the authors (J.N.) conducted a long-term follow-up telephone survey. Out of the 146 surveyed patients, 1 had passed away, and 38 others could not be contacted. The patients were interviewed using a standardized script regarding tape-related complications and whether they had undergone additional surgery for SUI. Patient-reported outcomes were evaluated by decision regret or satisfaction on a scale from 0 to 100 according to the Satisfaction with Decision Scale-Pelvic Floor Disorders [21,22]. A cure was defined as a satisfaction score ≥90. Improvement was defined as a satisfaction score of 61–89, and failure as a satisfaction score of ≤60. The primary outcomes were short- and long-term cure rates, complication rates, and patient-reported satisfaction. Patients who were categorized as cured or improved were compared to patients who were classified as failures.

Statistical analysis was performed using Student’s *t*-test for continuous data or Fisher’s exact test for categorical data. Data are summarized as mean ± standard deviation (S.D.) or percentage, according to the variables. Statistical tests were two-sided; a *p* value < 0.05 was considered statistically significant. SPSS software version 27 (IBM Corporation, Armonk, NY, USA) was used for the statistical analysis.

## 3. Results

A series of 146 consecutive patients (mean age 52.17 ± 11.77 years) who underwent inside-out transobturator Serasis^®^ MUS was analyzed. All patients experienced SUI as their primary symptom, and 30.8% of these patients had stress-predominant MUI. None of the patients had previously undergone anti-incontinence surgery. The demographic and clinical characteristics of the patients are presented in Table 1. Most patients also had concomitant stage I or II POP, and 12 (8.2%) had undergone a prior hysterectomy.

Intraoperative data are presented in Table 2. Most patients (80.14%) underwent combined inside-out transobturator Serasis^®^ MUS and anterior and/or posterior native tissue colporrhaphies. Five patients underwent combined MUS and apical repair. No significant intraoperative or early postoperative complications were recorded. The mean duration of the surgery was 26.03 ± 11.5 min, and the mean estimated blood loss was 31.13± 9.24 cc. All patients were discharged either on the day of their surgery or the following day (65.8% and 34.2%, respectively).

In total, 107 (73.3%) patients were available for the 10-year follow-up. Overall, 39 (26.7%) other patients were lost to follow-up, 20 (51.3%) of whom had MUI before the MUS surgery. There were no statistically significant differences regarding demographic, intraoperative, and early postoperative outcomes between patients who were lost to long-term follow-up and those who completed the study. Over the 10 years following surgery, 17 (15.9%) patients experienced persistent or recurrent SUI, and 9 (52.9%) of these patients underwent repeated MUS surgery. Two (1.9%) patients experienced tape erosion, and three (2.8%) patients reported de novo deep dyspareunia, all of whom underwent simultaneous colporrhaphies during the MUS procedure. None of the 107 available patients had any tape-related pain symptoms.

Patient-reported outcomes were assessed using a scale ranging from 0 to 100, with failure defined as a satisfaction score of 60 or lower. The failure rates observed at 4 months and 10 years postoperatively were 4.8% and 10.3%, respectively. At the 10-year follow-up, the primary cause of dissatisfaction was persistent OAB (seven patients; 63.6%). This was followed by SUI (three patients; 27.3%), de novo OAB (one patient; 9%), and POP (one patient; 9%). Conversely, among the 96 (89.7%) patients who were categorized as cured or improved (satisfaction score >60), only 5 (5.8%) patients experienced persistent OAB, and 4 (4.6%) other patients encountered de novo OAB. There were no cases of POP symptoms. The only factor determined to be statistically significant (*p* < 0.05) in predicting long-term subjective failure was the presence of OAB before MUS surgery. There were no significant differences observed in intraoperative outcomes or postoperative follow-up between the cohort of women who underwent concomitant colporrhaphies and those who did not (Table 3).

## 4. Discussion

Mid-urethral synthetic mesh slings have been established as the reference standard for surgical treatment of SUI since the procedure was introduced in 1995 [8,23]. Various types of commercially available mid-urethral slings (MUS) exist, distinguished by their methods of tape insertion and the materials utilized. Notable examples include Retropubic Slings (TVT^®^), Transobturator Slings (Monarc^®^/TVT-O^®^), and Single-Incision Mini-Slings (TVT-Secure^®^/Ajust^®^). The placement of the tape can be executed through the vaginal route (known as inside-out) or from the skin to the vagina (referred to as outside-in). Most commercially available MUSs are composed of lightweight, macroporous polypropylene mesh. Several meta-analyses have reported high success rates associated with MUS, and most international medical societies recommend this approach as the primary surgical option for addressing SUI [4,5,6]. A systematic review of 175 randomized controlled trials of 21,598 women analyzed 12-month outcomes of different surgical techniques for SUI [24]. Results of that systemic review show that pubovaginal fascial sling and retropubic MUS were more likely to cure SUI (89.4% and 89.1%, respectively), followed by open colposuspension (76.6%), transobturator MUS (64.1%), laparoscopic colposuspension (48.9%), single-incision sling (39.8%), and bladder neck needle suspension (26.9%). However, concerns about the safety and long-term efficacy of synthetic slings have been raised, mainly due to tape-associated erosions and pain [19,20]. The present study aimed to assess the long-term safety and efficacy of the Serasis^®^ transobturator MUS, which is a soft and partially absorbable tape.

Data on the long-term outcomes of MUS procedures are limited. In a recent systematic review that examined 44 studies involving 8218 women, only 22.7% of the studies provided long-term follow-up data extending to 10 years or more [25]. The results indicated that the overall re-operation rates at the 10-year follow-up varied: for trans-obturator MUS, the rates ranged from 5% to 15%, while for retro-pubic MUS, re-operation rates ranged from 2% to 17%. Similarly, other reviews, including the Cochrane and European Commission reviews, have not recommended one approach over the other [5,6,26,27,28]. Still, the National Institute for Health and Care Excellence (NICE) and the New Zealand Ministry of Health recommended that the trans-obturator approach only be offered in exceptional circumstances and following discussion in a multi-disciplinary or peer review forum [29,30].

While the overall rate of MUS tape-related complications is low, mesh erosions and chronic pelvic pain remain significant concerns. As a result, some medical authorities have issued warnings and restrictions on using MUS mesh implants. In 2019, the Royal College of Obstetrics and Gynecology (RCOG) published guidelines emphasizing heightened vigilance and restricting synthetic tape procedures for SUI [31]. The NHS Scottish committee review has raised similar concerns [20]. These concerns, along with media coverage and lawsuits against mesh manufacturers, have led to a worldwide decrease in the number of MUS procedures performed for SUI [32,33].

Serasis^®^ is a lightweight soft fabric trans-obturator MUS tape. The insertion of Serasis^®^ is considered less traumatic than other mid-urethral tapes. A previous study examined the safety and one-year outcomes of 50 patients who underwent transobturator MUS with the Serasis^®^ tape [34]. The primary outcome was early post-operative pain, and secondary outcomes were composed of SUI symptoms, pain, or other complications up to one year postoperatively. There were no cases of significant early postoperative pain, and, overall, 92% of patients were free of SUI symptoms at 1 year postoperatively. The authors concluded that the transobturator Serasis^®^ tape is safe and effective.

The present study evaluated the safety and 10-year outcomes of 146 consecutive patients who underwent the Serasis^®^ MUS procedure for SUI. Most patients also had concomitant native tissue colporrhaphies. The mean duration of surgery was 26.03 min, and the mean blood loss was 32.4 cc. All patients were discharged within a few hours after surgery or on the following day. No significant intraoperative or early postoperative complications were reported. During the 10-year follow-up, 17 (15.9%) patients reported symptoms of SUI, and half of them underwent a repeated MUS procedure. Rates of long-term tape-related complications were very low, with a tape erosion rate of only 1.9% and no reported tape-related pain symptoms. Erosion rates were based on the telephone interview. Patients were inquired about whether they had received a diagnosis of erosions or had undergone any procedures for their removal. All patients were subject to routine gynecological follow-up; therefore, we believe that the reported rates accurately reflect the true incidence of erosion. The long-term outcomes of this study demonstrate favorable results when compared to those associated with other conventional mid-urethral tape procedures. We therefore speculate that the mechanical properties of the tape and the partially absorbable material are among the factors that might be associated with these favorable observations. Additionally, 10.3% of the patients were categorized as a subjective failure, most of whom considered persistent OAB as the main reason for dissatisfaction. Establishing patient expectations for managing LUTS, including pharmacological interventions for OAB and surgical procedures to address SUI, is crucial. Effectively managing preoperative expectations increases patients’ satisfaction with the surgery.

The study has several strengths, including introducing a new type of trans-obturator, a partially absorbable tape with a soft texture that results in fewer tape-related complications, high patient satisfaction rates, and favorable long-term outcomes. Furthermore, this is the first report regarding the long-term subjective outcome of this unique tape. The study, however, encompasses several limitations that warrant consideration. Firstly, it is noteworthy that 26.7% of patients from the original cohort were unavailable for long-term follow-up, with a significant portion of these individuals presenting with preoperative MUI. This subgroup may represent a high-risk category for dissatisfaction, which could skew the study outcomes. Secondly, the analysis was conducted retrospectively and was carried out within a homogeneous population, all of whom were under the care of an experienced urogynecologist. Consequently, the findings of this study may not be generalizable to other demographics or less-experienced practitioners. Finally, the long-term follow-up relied solely on a telephone questionnaire without objective assessment tools, such as urodynamic studies. Nevertheless, we assert that subjective outcomes may possess greater clinical relevance, as other researchers in the field have suggested [4,35].

## 5. Conclusions

The transobturator Serasis^®^ MUS is a partially absorbable tape with a soft texture that has demonstrated both safety and long-term effectiveness in managing SUI. Its long-term outcomes are favorable and are associated with significantly fewer tape-related complications. The ongoing controversy regarding synthetic MUS highlights the importance of careful patient selection, thorough preoperative consultations, and continued research to enhance outcomes and address safety concerns.

## Figures and Tables

**Table 1:** Preoperative patients’ demographic, medical, and urogynecologic characteristics.

Mean + SD (Range), or N (%)	N = 146
Age (y)	52.17 ± 11.77 (38–77)
Parity	2.96 ± 1.25 (1–11)
Severe comorbidities *	0.73 ± 0.45 (0–7)
Prior hysterectomy	12 (8.2%)
Concomitant cystocele Stage 0 Stage I Stage II	23 (15.7%) 21 (14.4%) 102 (69.9%)
Concomitant apical prolapse Stage 0 Stage I Stage II	72 (49.3%) 69 (47.3%) 5 (3.4%)
Concomitant rectocele Stage 0 Stage I Stage II	32 (21.9%) 33 (22.6%) 81 (55.5%)
Overactive bladder	45 (30.8%)
Dyspareunia	19 (13.01%)

* Severe comorbidities: severe hypertension, diabetes, chronic obstructive pulmonary disease, ischemic heart disease, and congestive heart failure.

**Table 2:** Intraoperative data.

Mean + SD (Range), or N (%)	N = 146
Duration (min)	26.03 ± 11.5 (5–50)
Bleeding (cc)	31.13 ± 9.24 (20–65)
Concomitant Surgery None Anterior/posterior colporrhaphy Vaginal Hysterectomy Vaginal sacrospinous ligament fixation Cervical amputation (Manchester)	29 (19.86%) 117 (80.1%) 1 (0.7%) 1 (0.7%) 3 (2.05%)

**Table 3:** Early and long-term follow-up data.

Mean + SD, or N (%)	4 Months Follow-Up (N = 146)	10 Years Follow-Up (N = 107)
Satisfaction score ≥90 61–89 ≤60	93.7 ± 10.2 120 (82.2%) 19 (13%) 7 (4.8%)	90.3 ± 15.7 78 (72.9%) 18 (16.8%) 11 (10.3%)
Post-operative SUI re-operation	0 0	17 (15.9%) 9/17 (52.9%)
De Novo OAB	0	6 (5.6%)
Tape Erosions	0	2 (1.9%)
Dyspareunia Persistent De Novo	6 (4.1%) 6 (4.1%) 0	9 (8.4%) 6 (5.6%) 3 (2.8%)

## Data Availability

The original contributions presented in the study are included in the article; further inquiries can be directed to the corresponding authors.

## Abbreviations

The following abbreviations are used in this manuscript:

MUS	Mid Urethral Sling
SUI	Stress Urinary Incontinence
LUTS	Lower Urinary Tract Symptoms
OAB	Overactive bladder
MUI	Mixed Urinary Incontinence
